# Exogenous Ketones in Cardiovascular Disease and Diabetes: From Bench to Bedside

**DOI:** 10.3390/jcm13237391

**Published:** 2024-12-04

**Authors:** Urna Kansakar, Crystal Nieves Garcia, Gaetano Santulli, Jessica Gambardella, Pasquale Mone, Stanislovas S. Jankauskas, Angela Lombardi

**Affiliations:** 1 Department of Molecular Pharmacology, Division of Cardiology, Wilf Family Cardiovascular Research Institute, Einstein Institute for Neuroimmunology and Inflammation (INI), Albert Einstein College of Medicine, New York, NY 10461, USA; urna.kansakar@einsteinmed.edu (U.K.); gaetano.santulli@einsteinmed.edu (G.S.); gambardellajessica@gmail.com (J.G.); drpasquale.mone@gmail.com (P.M.); 2 Department of Medicine, Fleischer Institute for Diabetes and Metabolism (FIDAM), Einstein-Mount Sinai Diabetes Research Center (ES-DRC), Einstein Institute for Aging Research, Albert Einstein College of Medicine, New York, NY 10461, USA; crystal.nievesgarcia@einsteinmed.edu; 3 Department of Medicine and Health Sciences, University of Molise, 86100 Campobasso, Italy; 4 Casa di Cura Montevergine, 83013 Mercogliano, Avellino, Italy; 5 Department of Clinical and Molecular Medicine, School of Medicine and Psychology, Sapienza University, 00189 Rome, Italy

**Keywords:** exogenous ketones, BHB, diabetes, cardiovascular disease, SGLT2 inhibitors, metabolism, supplements

## Abstract

Ketone bodies are molecules produced from fatty acids in the liver that act as energy carriers to peripheral tissues when glucose levels are low. Carbohydrate- and calorie-restricted diets, known to increase the levels of circulating ketone bodies, have attracted significant attention in recent years due to their potential health benefits in several diseases. Specifically, increasing ketones through dietary modulation has been reported to be beneficial for cardiovascular health and to improve glucose homeostasis and insulin resistance. Interestingly, although excessive production of ketones may lead to life-threatening ketoacidosis in diabetic patients, mounting evidence suggests that modest levels of ketones play adaptive and beneficial roles in pancreatic beta cells, although the exact mechanisms are still unknown. Of note, Sodium-Glucose Transporter 2 (SGLT2) inhibitors have been shown to increase the levels of beta-hydroxybutyrate (BHB), the most abundant ketone circulating in the human body, which may play a pivotal role in mediating some of their protective effects in cardiovascular health and diabetes. This systematic review provides a comprehensive overview of the scientific literature and presents an analysis of the effects of ketone bodies on cardiovascular pathophysiology and pancreatic beta cell function. The evidence from both preclinical and clinical studies indicates that exogenous ketones may have significant beneficial effects on both cardiomyocytes and pancreatic beta cells, making them intriguing candidates for potential cardioprotective therapies and to preserve beta cell function in patients with diabetes.

## 1. Introduction

In recent years, tremendous interest has emerged in ketone metabolism due to numerous discoveries showing that ketone bodies not only serve as essential fuel sources but also act as signaling molecules, influencing cellular metabolism, inflammation, cell communication, and bioenergetics across nearly every organ [1]. Increasing evidence suggests that ketones may become a promising therapeutic target for a wide range of diseases, including cancer, obesity, aging, neurodegeneration, inflammatory disorders, diabetes, and heart failure [2,3,4]. While ketone bodies have historically been viewed with fear by clinicians, particularly due to their association with diabetic ketoacidosis and severe illness, the perception of these molecules has evolved significantly. Initially seen as metabolic byproducts, it became clear that ketones are crucial alternative energy substrates, particularly for the brain during periods of starvation or carbohydrate deprivation. Furthermore, the role of ketones has expanded beyond energy provision to include metabolic signaling, with emerging research highlighting their involvement in various tissues, including immune cells in the visceral adipose tissue [5] and intestinal cells [6].

The term “ketone bodies” usually implies three main products of ketogenesis: β-hydroxybutyrate (BHB), acetoacetate (AcAc), and acetone. Of note, amino acids like lysine and leucine are regarded as strictly ketogenic amino acids, providing carbon units for ketone bodies synthesis in the liver [7,8,9]. BHB is the most abundant ketone body and its levels in circulation change much faster in response to metabolic changes compared to AcAc or acetone [1,10].

Recent data from in vivo, ex vivo, and in vitro experiments, as well as clinical trials, suggest that ketones are attractive molecules that may act synergistically with pharmacotherapy and can lead to new strategies to preserve cardiac and beta cell function.

## 2. Ketones as Prognostic Markers in Cardiovascular Disease and Diabetes: Friend or Foe?

Alterations in cardiac energy metabolism play a central role in the mechanism of heart failure (HF) [11]. In the heart, ketone bodies may play a dual role, providing energy and signaling functions in both healthy and diseased states [12,13]. Basic research strongly conjectures that BHB supports heart function in disease [12,13].

Real-world data show that higher levels of BHB are associated with a higher risk of HF. In the elderly population (70–82 years) from the PROspective Study of Pravastatin in the Elderly at Risk (PROSPER) cohort study, increased serum levels of BHB were a predictive marker of HF [14]. The Prevention of Renal and Vascular End-stage Disease (PREVEND) study enrolled 8592 male and female residents in the city of Groningen (the Netherlands), between the ages of 27 and 75 years, with albuminuria [15]. Analysis of this population revealed sex differences in BHB’s association with HF incidence [16]. During the 8.2-year follow-up period, BHB was a strong predictor of HF and heart failure with reduced ejection fraction (HFrEF) in women, but not in men, with hazard ratios of 2.12 and 4.75 in women against 1.17 and 1.09 in men (for HF and HFrEF, respectively). Higher levels of BHB were predictive for HF independently from the body mass index (BMI), type 2 diabetes (T2D), hypertension, myocardial infarction, dyslipidemia, kidney function, smoking, and alcohol consumption. However, when adjusted for a history of atrial fibrillation, heart rate, and NT-proBN levels, the effect of BHB became non-significant [16]. In another small study, levels of BHB were found to be higher in HF patients compared to healthy counterparts [17]

Studies in HF populations also confirmed a positive correlation between circulating BHB and negative outcomes. In a prospective, single-center, observational study with a total of 152 hospitalized patients with acute HF, BHB predicted 3-month mortality. The hazard ratio was 2.15 even after adjustment for age, sex, BMI, T2D, kidney function, arterial pressure, LDL-cholesterol, and NT-proBNP [18]. Post-analysis of the LIVE trial (clinicaltrials.gov identifier: NCT01472640), which evaluated the effect of glucagon-like peptide-1 analog liraglutide on HF in T2D patients [19], found reduced 9-year mortality in patients with low plasma BHB (<0.059 mM) [20]. BHB predicted both the development of arrhythmogenic cardiomyopathy (AC) and the incidence of major adverse cardiovascular events in AC patients [21]. In a larger study that enrolled 867 HFrEF patients, high BHB predicted a higher incidence of composite events (including death, urgent transplantation, or left ventricular assistance device implantation) during an 8-year observation period [22]. Interestingly, high levels of BHB were also associated with a higher prevalence of diabetes and higher levels of free fatty acids (FFAs) [20,22]. Moreover, high levels of FFAs predicted high BHB levels in a multi-variable analysis [20]. Composite events were more frequent in patients with high FFAs and low BHB than in those with low FFAs and high BHB [22]. These data strongly suggest the absence of a causative connection between elevated BHB and HF.

Data from myocardial infarction (MI) populations confirms the idea that elevated BHB is not detrimental to the heart. In a GIPS-III trial (NCT01217307), blood samples were obtained from 360 non-diabetic patients with ST-Segment Elevation Myocardial Infarction (STEMI) before percutaneous coronary intervention (PCI) as well as 24 h and 4 months after [23]. BHB levels before PCI were significantly higher than 24 h after, and 24 h levels were higher than at the 4-month time point. Moreover, 24 h levels of BHB positively correlated with myocardial infarct size and negatively with left ventricular ejection fraction (LVEF) [24]. A smaller study in 59 STEMI patients confirmed a decrease in BHB levels after PCI [25]. These data suggest that an increase in BHB is a consequence of cardiac injury and/or dysfunction, rather than their mediator. Moreover, recent data suggest that increased production of BHB might represent a protective mechanism. For instance, in the EMMY trial (NCT03087773) cohort (476 acute MI patients), high baseline levels of BHB at admission were associated with worse cardiac function at the end of the 26-week follow-up period [26]. However, in the same cohort, an increase in BHB levels during the observational period was associated with an improvement in cardiac parameters [26].

Plausibly, ketonemia in HF and MI patients results from increased ketogenesis in the heart. Early studies demonstrated that in HF patients, a fasting period elicits higher ketonemia than in healthy participants [27]. In a recent comprehensive study, Jiang-Ping Song, Liang Chen, and co-authors demonstrated that, at least in AC, ketonemia is a result of cardiac BHB production [21]. They performed proteomic and metabolomic analyses of heart explants from AC patients and healthy volunteers and discovered that the expression of enzymes involved in BHB synthesis was increased in AC samples. Expression of BHB transporters mediating both uptake and efflux of the compound was also upregulated. It was accompanied by higher plasma levels of BHB in AC patients compared to healthy volunteers [21]. However, definitive results came from 13 AC patients who underwent heart transplantation. In all 13 patients, circulating BHB was drastically decreased after transplantation compared to pre-surgery levels. At the same time, levels of NEFAs (non-esterified fatty acids), serving as a precursor for ketone bodies, remained unchanged in these patients, suggesting that decreased BHB production was not the result of limited substrate supply [21].

Importantly, in all the above-mentioned studies, the increase in serum concentration of BHB in a predominant number of patients remained in the normal range (0.1–0.3 mM), with only a few participants exceeding 0.5 mM. These findings rule out the possibility of ketoacidosis being the mediator of those effects and suggests that BHB acts as a signaling metabolite fine-tuning cardiac function.

Increased levels of BHB also predict a higher incidence of diabetes [28]. The research group of A.V. Ahola-Olli and P. Würtz analyzed a large Finish cohort of 11,896 individuals pooled from four different studies (namely YFS, FINRISK-1997, DILGOM, and NFBC). Participants were younger than 45 years old and diabetes-free. Increased plasma levels of BHB were associated with a higher risk of T2D development during 10 years of follow-up [29]. Of note, the analysis of a Dutch cohort of 3307 participants in the PREVEND study confirmed these results. Interestingly, BHB was associated with higher risk of T2D development independently from age, sex, BMI, dyslipidemia, insulin resistance, or renal function. Moreover, T2D incidence increased in a dose-dependent manner when individuals were stratified according to BHB concentration [30]. The METSIM (Metabolic Syndrome in Men) study including 4335 healthy or newly diagnosed diabetic participants found higher BHB levels in patients with high fasting glucose levels [31]. Another pooled study evaluated 1623 clinically healthy individuals aged 30–60 years old from The Relationship between Insulin Sensitivity and Cardiovascular Disease Study (RISC Study) and The Diabetes Mellitus and Vascular Health Initiative (DMVhi) screening study. In this study, investigators found a statistically significant positive correlation between circulating BHB levels and impaired glucose tolerance [32]. Furthermore, BHB also predicted the development of T2D in obese patients [33].

A study of a small cohort of pregnant women identified plasma metabolites that were able to discriminate healthy pregnant women from those with gestational diabetes. BHB was the most highly upregulated metabolite in the gestational diabetes group both in the second and third trimesters [34]. Moreover, higher levels of BHB in the second trimester and 3 months postpartum predicted the development of T2D during the 2 years of follow-up [34]. Another smaller study demonstrated that BHB levels were able to predict the development of gestational diabetes in obese women [31].

However, like in HF, increased BHB levels are most likely to be a consequence of T2D or even an adaptive response. Despite BHB being positively correlated in two different studies with the risk of T2D development or the presence of hyperglycemia, in the same cohorts, BHB levels correlated positively with better insulin sensitivity [29,35]. More conclusive evidence comes from the study of 369 newly diagnosed drug-naïve T2D patients. In this study, high levels of BHB were associated with better response to the treatment at 3 and 6 months of follow-up [36]. These results are in contradiction with a study of patients receiving metabolic surgery. Higher circulating BHB levels were a negative predictor of response during 3 months of follow-up [37]. Moreover, in this study, plasma BHB levels demonstrated a strong negative correlation with HbAc1 [37]. However, this discrepancy may be explained by the fact that individuals in the non-responder group had a longer duration of diabetes.

## 3. Ketones and Cardiovascular Disease

### 3.1. Animal Studies

Different animal models of HF provide compelling evidence of the therapeutic potential of BHB.

In a two-hit (high-fat diet (HFD) + L-NAME) rat model of HF with preserved ejection fraction (HFpEF), recapitulating the cardiac dysfunction in diabetes, both circulating and myocardial levels of BHB gradually decreased during the disease course. Moreover, expression of a key enzyme for BHB metabolism—D-beta-hydroxybutyrate dehydrogenase (BDH1)—was also lower in the heart of HFpEF rats [38]. A Sodium-Glucose Transporter 2 inhibitor (iSGLT2), Dapagliflozin (DAPA), restored BHB levels and mitigated diastolic dysfunction, myocardial fibrosis, and hypertrophy. These effects were accompanied by restoration of the normal level of mitochondrial electron transport chain proteins abundancy [38]. Interestingly, DAPA did not restore BDH1 levels, but in contrast, further decreased it. This may suggest that restoration of BHB levels was due to decreased utilization of BHB, and hence BHB protected the heart not as an energic substrate but as a signaling molecule [1,38]. However, this study did not employ pharmacological or genetic inhibitory approaches to tell correlative and causative effects apart. Effects of HF and DAPA on BHB utilizations remain debatable, as in the transverse aortic constriction (TAC) model higher BHB oxidation was detected in diseased hearts, and DAPA further increased it [39]. Despite being a glucose-lowering drug, DAPA did not decrease glucose utilization in the heart, additionally supporting the hypothesis that the promotion of ketosis is the primary mechanism of iSGLT2’s cardioprotection [39]. And indeed, in a more severe model of HFpEF combining aging, HFD, and desoxycorticosterone pivalate, BHB supplementation in the form of ketone ester and the iSGLT2 Empagliflozin (EMPA) demonstrated the comparable protective effect [40]. Furthermore, in a two-hit model of HFpEF, BHB treatment alone was able to restore normal diastolic function [41].

Recently, our group demonstrated that in a murine model of post-ischemic HF a chow supplemented with BHB mitigated myocardial lesions and cardiac dysfunction [42]. BHB also preserved mitochondrial mass and function. The effect of BHB on mitochondria was found to be underlined by the epigenetic control of Peroxisome proliferator-activated receptor Gamma Coactivator 1-alpha (PGC-1α) expression. Importantly, these BHB-controlled epigenetic changes were detected both in the murine model of HF and human heart samples from patients with HF [42]. In contrast to BHB supplementation, the ketogenic diet did not exert protective action in a similar model of HF [43] and impaired cardiac energetic flexibility and efficiency reducing glucose oxidation, even under insulin injection [43]; it also failed to improve heart function in another model combining TAC and myocardial infarction [44]. However, in the same model, cardiomyocyte-specific KO of BDH1 exacerbated HF, demonstrating the instrumental role of BHB utilization under stress conditions [44]. Intriguingly, bioenergetic effects of BHB were similar to those of a ketogenic diet: reduced glucose utilization and higher dependence on fatty acid utilization. However, BHB infusion retarded the progression of tachycardia-induced HF, again highlighting the non-energetic effects of BHB [44].

BHB was demonstrated to elicit immediate protection in the model of cardiac ischemia—reperfusion (I/R) injury. Single intraperitoneal injection of BHB immediately after 45 min ischemia in one study or continuous releases of BHB from an osmotic pump after 30 min ischemia reduced myocardial infarction and improved ejection fraction, as measured after 24 h of reperfusion [25,45]. The protective effect of BHB vanished in Atg7-KO mice, highlighting the pivotal role of autophagy in BHB-mediated cardioprotection. And indeed, the authors demonstrated an increase in autophagosome flux after BHB administration [25,45]. In line with Gambardella’s findings, BHB rescued a decrease in mitochondrial mass after I/R [25]. BHB also mitigated cardiac and mitochondrial dysfunction in the pig model of heart transplantation [46]. In line with the HF results, mice with MI that were fed a ketogenic diet showed worsened cardiac dysfunction compared to MI mice on a control chow diet [47].

BHB also exhibited a therapeutic potential in other models of cardiovascular pathologies. In a recent study, Lan and colleagues demonstrated that the supplementation with 1,3-butanediol (1,3-B), a precursor of BHB, helped reduce aortic calcification in rats with chronic kidney disease and in mice treated with excessive vitamin D3. Moreover, their research revealed that BHB prevents vascular calcification by influencing the NF-KB signaling pathway through HDAC9 modulation [48]. Similarly, in another study, Chakraborty et al. showed that 1,3-B supplementation increased BHB levels and offered protection to mice against hypertension and kidney inflammation. However, they also found that a high-salt diet led to a reduction in BHB levels [49]. In streptozotocin (STZ)-induced diabetes mellitus rats, the ketogenic diet improved cardiac function and decreased myocardial fibrosis and apoptosis as compared to the control mice that were fed a normal diet [50]. The latest results may sound contradictory to the absence of the protective effect of the ketogenic diet in models of HF. However, in STZ-model of diabetes, pancreatic beta-cells are destroyed and insulin production vanishes. Hence, the protective effect of the ketogenic diet might be attributed to either BHB production or to reduced carbohydrate intake.

### 3.2. Human Studies

Stimulation of ketogenesis to harness potential BHB cardio protection is an objectionable approach due to the risk of ketoacidosis. Finding relatively safe physiological ways to upregulate intrinsic BHB production is crucial, as fasting or ketogenic diets have many limitations related to compliance and specific dietary requirements in some of the pathologies accompanying HF. This makes a case for utilizing exogenous BHB. The ability of BHB to modulate cardiac function has been demonstrated in healthy young volunteers. Orally delivered ketone ester (releasing BHB in the organism) increased cardiac performance and decreased systemic vascular resistance [51,52]. Intravenous (i.v.) administration of BHB increased heart rate and myocardial blood flow [53].

In a small, acute, but well-designed study, exogenous BHB exerted beneficial hemodynamic effects in patients with HFrEF [54]. The study enrolled 24 patients with LVEF ≤40% and 10 age-matched healthy volunteers. Participants received three different doses of BHB (one supraphysiological and two physiological) via i.v. infusion, and various cardiac function parameters were measured 3 h later. In the treatment arm (all doses), cardiac output, stroke volume, LVEF, heart rate, and oxygen consumption were higher compared to placebo. Systemic and pulmonary vascular resistance and mean pulmonary pressure were lower. In age-matched healthy participants, BHB exerted similar effects. Dynamic measurement of blood levels of BHB and cardiac output demonstrated a strong positive correlation between these parameters in time. BHB infusion increased oxygen consumption and cardiac work proportionally, leaving myocardial external energy efficiency unaltered [54]. This means that coupling between myocardial energy generation and cardiac work was not compromised, despite decreased vascular resistance. This is important for safety considerations. Reduced myocardial external energy efficiency is a powerful clinical predictor for cardiac death in patients with HF [55].

In an elegant study published in 2023, Nigopan Gopalasingam and co-authors from Aarhus University Hospital aimed to delineate the signaling mechanism of BHB [56]. The biological activity of BHB is mediated through different mechanisms, including direct inhibition of histone deacetylase, β-hydroxybutyrylation of proteins, facilitation of oxidative phosphorylation in mitochondria, and stimulation of two plasmalemma receptors—Hydroxycarboxylic acid receptor 2 (HCA2) and Free fatty acid receptor 3 (FFAR3 or GPR41) [1]. The Gopalasingam group focused on HCA2. This G-protein-coupled receptor has two ligands: niacin and BHB [57,58]. In animal studies, stimulation of HCA2 by BHB exerted two effects important for cardiovascular health. First, it downregulated lipolysis in adipocytes, thus reducing their levels in circulation [58]. Moreover, the second HCA2 ligand, niacin, is one of the most effective lipid-modifying drugs [59]. The second effect of HCA2 activation is the attenuation of the L-type Ca^2+^ current (I_CaL_) [60]. I_CaL_ plays a critical role in cardiomyocyte contraction. However, its activity is increased in hypertrophic and failing hearts and plausibly facilitates arrhythmogenicity [61]. Additionally, stimulation of HCA2 with niacin triggers the production of vasoactive prostanoids, modifying the course of congestive HF [62,63,64]. Moreover, the BHB effect on the heart could be mediated in an HCA2-independent manner. To distinguish between these signaling mechanisms, researchers compared the effects of BHB and niacin in the presence or absence of aspirin, which blocks prostanoid synthesis [56]. The study was conducted on 28 patients with HHrEF. All patients were monitored with pulmonary artery catheter measurements, echocardiography, and blood samples at baseline and hourly for a 6 h period after drug injection. BHB increased cardiac output and diminished FFA levels both in the presence and absence of aspirin, meaning that BHB effects are independent of prostaglandin/prostacyclin synthesis [56]. Indeed, BHB treatment did not affect the concentration of prostaglandin D2. Interestingly, niacin as well as BHB lowered serum FFA but failed to increase cardiac output [56]. These data strongly suggest that the cardiac effects of BHB are not mediated by HCA2 signaling. This study also possesses clinical value. In addition to cardiac output, BHB ameliorated a number of other cardiac function parameters, including stroke volume, LVEF, left ventricular end-diastolic volume, venous saturation, systolic mitral plane peak excursion velocity, tricuspid annular peak systolic excursion, and oxygen saturation. BHB decreased systemic vascular resistance and slightly, but statistically significantly, reduced mean arterial pressure [56].

Two studies conducted by the same team from Aarhus University in Denmark investigated the effects of a longer oral uptake of BHB [65]. Both studies followed a similar randomized, double-blind crossover design. Twenty-four patients were randomized into two groups that received either ketone ester or isocaloric comparator for 14 days, followed by a 14-day washout period, after which treatment and placebo groups were switched. Participants were analyzed at the end of the 14-day treatment period. One study evaluated non-diabetic HF patients while the second evaluated HF patients with T2D. In nondiabetic HFrEF patients, BHB exerted a marked beneficial effect improving all parameters of cardiac function, including the most important one—stroke volume and LVEF. Systemic vascular resistance was also ameliorated in this cohort [65]. Results in diabetic patients were much more modest. BHB intake improved only cardiac output and average early diastolic velocity and diminished central vascular resistance. Changes in other cardiac parameters were statistically non-significant [66].

The effect of exogenous BHB was also tested in patients with cardiogenic shock KETO-SHOCK1 (NCT04642768) [67]. Half of the patients enrolled in the study had cardiogenic shock due to acute MI. Intravenous infusion of ketone ester for 3 h robustly improved cardiac function compared to the placebo arm [67]. Although the results are promising, the design of the study was not intended to analyze mortality, which is the most important outcome.

The small clinical trials described above make a strong case for exogenous BHB to be considered a promising drug candidate for HF treatment. These short-term human studies are supported by basic research, demonstrating the robust beneficial effect of exogenous BHB long-term treatment in different animal models of HF [42,68,69].

Dyslipidemia plays a highly determining role in cardiovascular health [70]. Being the ligand of HCA2, one may expect BHB to exert an FFA lowering effect, in similarity with the potent action of an HCA2 second ligand—niacin [59]. Indeed, acute studies support this premise. Administration of exogenous BHB decreased FFA in blood in the acute period 1–3 h after infusion [53,54,56]. However, long-term observations yielded the opposite results. In the CANVAS (NCT01032629) and CANVAS-R (NCT01989754) trial cohorts, levels of serum FFA significantly correlated with BHB [71]. In two small studies done in diabetic patients with inhibitors of sodium-glucose transporter 2 (iSGLT2), 1 month of EMPA or 2 weeks of DAPA intake augmented both BHB and FFA levels [72,73]. The discrepancy between acute and long-term studies might be explained by the fact that long-term studies were done in the diabetic population, and T2D is strongly associated with dyslipidemia. This notion could be supported by findings in trials that did not target diabetes specifically. In the PREVEND cohort, high levels of BHB predicted T2D development [30]. A study in HFrEF patients observed simultaneously a strong positive correlation between BHB and FFA and between high levels of BHB and diabetes [22]. Another HF trial demonstrated that high levels of FFA predicted high BHB levels in multi-variable analysis [20]. This data shows that a positive correlation between BHB and diabetes may obscure the FFA-lowering effect of BHB and further research is required.

There is a significant gap in the literature regarding the impact of ketone supplements on blood pressure in humans, particularly in at-risk populations like individuals with hypertension and older adults. Furthermore, additional research is needed to explore the effects of ketone supplements in hypertension [74].

Nielsen et al. (NCT04615754) enrolled 10 patients with pulmonary arterial hypertension (PAH) and 10 with chronic thromboembolic pulmonary hypertension (CTEPH), all having residual pulmonary hypertension. A randomized crossover study was conducted with BHB infusion or placebo. Infusion of BHB raised circulating BHB levels, which resulted in a 27% improvement in cardiac output and a 13% increase in right ventricular systolic velocity. Pulmonary vascular resistance decreased by 18%. No significant difference in response was observed between the PAH and CTEPH groups [75]. In a randomized, double-blinded trial (NCT03452761), 23 participants received either exogenous ketone salt supplementation or placebo two times per day for 6 weeks. Chronic ketone supplementation significantly increased blood ketone levels 30 and 60 min after supplementation and may reduce systolic blood pressure reduction. Another randomized controlled crossover trial (NCT03461068) enrolled 15 participants with obesity. Participants underwent an overnight fast and then consumed either a ketone monoester (KE) drink containing BHB or a taste-matched placebo 30 min prior to performing a 75 g oral glucose tolerance test (OGTT). There were no differences in the area under the curve (AUC) for triglycerides, C-peptides, or insulin following consumption of the KE drink. However, the KE drink led to a reduction in mean arterial blood pressure and an increase in heart rate [76]. A clinical trial (NCT03073356) enrolled patients with HF and reduced ejection fraction. Infusion of BHB resulted in a 40% increase in cardiac output (2 L/min) and an 8% improvement in left ventricular ejection fraction. These changes were accompanied by vasodilation, with systemic and pulmonary blood pressures remaining stable [54].

## 4. Ketones and Diabetes

Ketone bodies have long been known to improve the survival of nerve cells, and interestingly, beta cells share significant common genetic and physiological characteristics with neurons [77,78]. Although excessive production of ketones may cause ketoacidosis in diabetic patients, recent studies demonstrate that modest levels of ketones play adaptive and positive roles [79]. Moreover, chronic treatment with BHB improves beta cell function and mitochondrial health in both mice and humans, helps in the recovery from lipotoxic stress, enhances the expression of key genes that are involved in insulin production [80] and might impact viral infection of pancreatic beta cells [81].

### 4.1. Animal Studies

In recent years, many studies have been conducted exploring the role of ketone bodies in the progression of diabetes. These studies have used several murine models and cell lines to test the potentially therapeutic effects of ketogenic diets and exogenous ketone supplementation on the development of diabetes and its associated pathologies.

Carbohydrate- and calorie-restricted diets have been successfully used to improve beta cell function and insulin resistance, suggesting that states of ketogenesis may have beneficial effects in T2D [82]. In a study by Zhang et al., mice with T2D were given water treated with 1,3-B. The treatment with medicated water reduced fasting blood glucose levels, improved glucose tolerance, and ameliorated insulin resistance through HCA2 [83].

In a different study, a mouse model of T2D was used to explore the effects of a ketogenic diet on muscle mass and function. The diabetic condition caused a reduction in muscle mass and grip strength, but being placed on a ketogenic diet for 14 weeks ameliorated these effects. Recent studies also demonstrated that following the ketogenic diet intervention, the expression of nucleotide-binding and oligomerization domain-like receptor family pyrin domain-containing 3 (NLRP3) inflammasome markers and endoplasmic reticulum (ER)-stress-related markers in the muscles of the mice were positively modulated [84]. These findings demonstrate how increasing BHB levels—in this case, by inducing ketosis through feeding mice a ketogenic diet—can improve glucose control and alleviate symptoms of diabetes. Similarly, in a study by Trotta et al., T2D was induced in C57BL6J mice, which caused upregulation of retinal HCA2 receptors, elevation of several retinal ER stress markers, and increased levels of NLRP3 inflammasome activity markers and proinflammatory cytokines. The mice were then treated systematically with intraperitoneal injections of BHB, which resulted in significantly reduced levels of proinflammatory cytokines and retinal ER stress and NLRP3 activity markers. The investigators concluded that when activated by BHB, HCA2 receptors exert an anti-inflammatory effect on retinal damage induced by diabetes by reducing NLRP3 inflammasome activity and proinflammatory cytokine levels [85]. Furthermore, it has been demonstrated that BHB improves acute pancreatitis in male Wistar albino rats by inhibiting the NLRP3 inflammasome pathway [86].

The role of the mitochondrial β-oxidation enzyme HADHA in hepatic glucagon response was investigated by Pan and colleagues. Their study found that in high-fat-diet fed mice, HADHA overexpression improved metabolic disorders, and this effect was negated by knockdown of a BHB-producing enzyme. They concluded that BHB is responsible for HADHA’s inhibitory effect on hepatic glucagon response, again highlighting BHB elevation as a promising strategy for treating T2D [87].

Of note, BHB targets the glycogen synthase kinase 3β-controlled nuclear factor-erythroid-2-related factor 2 (Nrf2) antioxidant response and can diminish podocyte senescence and injury caused by T2D. This effect was observed both in vitro—in podocytes exposed to diabetic milieu—and in vivo—in a murine model of diabetic kidney disease (DKD) [88].

Also, a study by Wan and colleagues examined the role of the BDH1-mediated BHB metabolic pathway in the pathogenesis of DKD. They found that in mouse models of DKD and high glucose HK-2 cells, BDH1 is downregulated. However, induction of BDH1 renal expression reversed fibrosis, inflammation, and apoptosis in the kidneys of this DKD mouse model, and BDH1 overexpression or BHB treatment protected HK-2 cells from glucotoxicity. These results highlight the potential of ketogenic diets or BHB supplementation as therapeutic approaches to treating DKD progression [89].

Furthermore, Oka et al. explored the role of BHB in the regulation of Trx1, an antioxidant that aids in protecting the heart during the development of diabetic cardiomyopathy. Their results demonstrated that BHB enhances antioxidant defense in cardiomyocytes through inhibition of HDAC1 and increased stabilization of Trx1, suggesting that increased levels of ketone bodies could protect the hearts of T2D patients against diabetic cardiomyopathy [90].

Interestingly, in mice, the induction of an endogenous ketogenesis deficiency by a whole-body *Hmgcs2* deletion shortens the life span, but this effect can be prevented using ketone supplementation with 1,3-B. When aged knockout mice were fed a diet supplemented with 1,3-B, their life spans were extended and mortality rates decreased, thus highlighting the significance of ketone bodies to the process of aging and the metabolic diseases with which it is associated [91].

It has also been shown that BHB offers protective effects in the pancreas and tissues against glutathione (GSH)-deficiency-caused ferroptosis in acute liver failure (ALF) mouse models [92]. Several other studies have concluded that BHB influences pancreatic islet function and hormone regulation. For instance, acute treatment with R-βHB enantiomer of C57BL/6J mouse islets increases insulin secretion and decreases glucagon secretion at physiological glucose levels. This suggests that BHB may contribute to promoting islet cell health and survival [93].

### 4.2. Human Studies

Generally, exogenous ketone supplementation, particularly BHB, may offer therapeutic benefits for T2D by reducing hyperglycemia, inflammation, and oxidative stress [94]. Several clinical trials have been conducted to explore whether the therapeutic benefits of ketogenic diets and exogenous ketone supplementation that have been demonstrated in murine and cell models can also be observed in healthy individuals, as well as patients living with or at risk of diabetes.

In a clinical study with women on long-standing ketosis, Cooper and collaborators found that sustained ketosis can alleviate hyperinsulinemia without diminishing metabolic flexibility, which may provide several benefits in counteracting chronic diseases and biological aging [95].

Moreover, Yu et al. investigated the effects of ketone supplementation on blood BHB, glucose, and insulin levels in a population with obesity and prediabetes. Their studies found that BHB exhibits a glucose-lowering effect without increasing insulin, which points to the potential therapeutic benefits of exogenous ketone supplementation for individuals who have or are at risk of T2D [96].

In young individuals, consumption of a ketone monoester supplement 30 min prior to a glucose tolerance test reduced both glycemic response and insulin sensitivity markers without altering insulin secretion. These results point to the future therapeutic potential of ketone supplements as glucose-lowering agents in the management of metabolic diseases [97].

A recent study investigated whether BHB can suppress pro-inflammatory cytokine secretion: liposaccharide-stimulated leukocytes from overnight-fasted adults at risk for T2D were treated with BHB, and results showed that BHB treatment suppressed secretion of the pro-inflammatory cytokines IL-1β, TNF-α, and IL-16 while increasing secretion of the anti-inflammatory cytokines IL-1Ra and IL-10 [98].

The effects of two novel ketone supplements—with slightly different chemical makeups from ketone monoesters—on blood BHB and glucose levels were investigated in healthy individuals. It was found that following ingestion, BHB levels were raised and glucose levels decreased, comparable to that of an already established ketone monoester supplement [99].

Soto-Mota et al. investigated the effects of L-alanine supplementation on the glucose-lowering effect of exogenous ketone supplementation, as L-alanine is a gluconeogenic substrate secreted at higher levels in patients with T2D. The study found that in healthy patients supplemented with both exogenous ketones and L-alanine after 24 h of fasting, the ketosis-induced drop in blood glucose levels was significantly reduced. The authors concluded that the glucose-lowering effect of increased blood BHB levels is partially due to BHB decreasing the availability of L-alanine as a substrate for gluconeogenesis [100].

While the therapeutic benefits of exogenous ketone monoesters on blood glucose levels have been demonstrated, downsides such as unpleasant taste and gastrointestinal discomfort highlight the need for novel supplements. Moreover, BHB also appears to support pancreatic ductal adenocarcinoma (PDA) growth and metastasis by serving as an alternative energy source, with the enzyme HMG-CoA lyase (HMGCL) playing a key role in ketogenesis [101].

These studies demonstrate promise in using ketogenic diets and exogenous ketone supplementation to treat diabetes, but more studies need to be conducted to fully elucidate the mechanisms responsible for the therapeutic effects that have been observed thus far.

## 5. Ketones and SGLT2 Inhibitors

iSGLT2s, also known as gliflozins or flozins, are a class of medications that are widely used in the treatment of T2D due to their ability to lower blood glucose levels [102,103,104,105,106].

SGLT2 is predominantly expressed in the renal tubules of the kidneys and is responsible for reabsorbing 90% of glucose. As a result, any excess glucose is eliminated through urine [107,108,109,110,111,112].

Primarily developed as anti-diabetic medication, iSGLT2 recently became a pharmacological blockbuster after the discovery of their diabetes-independent cardioprotective effect [113,114]. The mechanism of cardio protection elicited by iSGLT2s remains elusive. In this section, we will describe animal and human studies analyzing the role of BHB in iSGLT2 protection against cardiovascular disease (CVD).

The iSGLT2s that are FDA-approved for the treatment of T2D patients include canagliflozin (CANA), DAPA, EMPA, and Ertugliflozin (ERTU).

### 5.1. SGLT2 Inhibitors Cardioprotective Action: Animal Studies

A major effect of iSGLT2s observed in animal studies is the cardiac remodeling effect. In one of the very first rodent model studies by Byrne et al., mice with induced HF treated for 14 days with EMPA were significantly protected from the progressive deterioration of cardiac function characterizing vehicle-treated mice [115]. Further studies investigated potential pathways influencing iSGLT2s’ cardiac remodeling effects in rodents, suggesting mechanisms involving anti-metabolic and anti-inflammatory effects [116], protection from oxidative stress [117], and a possible microbiota involvement [118]. iSGLT2s improved exercise endurance capacity in HF mice, without affecting cardiac functions post-MI [119], and increased lipolysis [120], through a mechanism suggesting the activation of the AMPK pathway [121] and the increase in BHB levels [122].

Due to the pig model’s suitability to elucidate metabolic queries of the human metabolism, there are a few iSGLT2s pig model studies. The findings from these analyses revolve mainly around metabolic alterations following MI and iSGLT2s treatment, with a focus on the cardiac remodeling. Baker et al. investigated the impact of canagliflozin (CANA) on contractile function, substrate utilization, and efficiency before and during regional MI in normal, metabolically healthy pigs, demonstrating a preservation of cardiac contractile function and efficiency during acute regional MI through acute effects on cardiac volume regulation [123]. Moreover, in another study, DAPA decreased both systolic and diastolic blood pressure and alleviated the sympathetic tension in the aorta [124]. Similarly, EMPA improved myocardial energetics and cardiac functions through the shift in myocardial fuel metabolism away from glucose and towards cardiac utilization of ketone bodies, free fatty acids, and branched-chain amino acids [125].

A study conducted by Baartscheer et al. demonstrated a clear effect of EMPA in rabbits, resulting in the improvement of heart arrhythmia, oxidative stress, and heart failure [126]. Another group in a more recent study showed that treatment with DAPA reduces the development of atherosclerotic lesions in a normoglycemic rabbit model, suppressing lipid accumulation, intimal proliferation, and pro-inflammatory marker levels, suggesting beneficial anti-atherosclerotic effects of iSGLT2s [127].

Currently one zebrafish animal model study of iSGLT2s’ cardioprotective effects has been conducted: in this study, Shi et al. examined biomarker changes elicited by EMPA in a model of heart failure. Interestingly, HF was completely reversed in zebrafish embryos exposed to aristolochic acid; finally, EMPA also decreased the expression of pro inflammatory cytokines including IL-1β [128].

### 5.2. SGLT2 Inhibitors Cardioprotective Action: Human Studies

Mounting evidence provides incontestable support for SGLT2 inhibitors’ ability to promote ketogenesis. On top of anecdotal reports of euglycemic ketoacidosis caused by iSGLT2 [129,130,131], an increase in physiological concentrations of BHB have been recorded in several clinical trials. In a small trial evaluating the effects of DAPA in prediabetic insulin-resistant individuals, BHB levels were higher in the treatment group compared to the placebo [72]. In a trial on diabetic patients with CVD risk, a 30-day course of EMPA or glimepiride significantly increased BHB levels compared to the sulfonylurea-treated group [73]. The comparison to another anti-diabetic drug rather than a placebo is the great advantage of this study. Such design allows an assessment of whether BHB upregulation is specific for iSGLT2 or results from the glucose lowering effect. Indeed, both treatments decreased levels of glycated albumin during the trial period, suggesting that increased ketogenesis is the unique feature of gliflozin-class medications [73]. Treatment with metformin in another study was not associated with changes in BHB levels, further supporting this notion [24]. In the trial “*DAPA Effects on Biomarkers, Symptoms and Functional Status in Patients with HF With Reduced Ejection Fraction*” (DEFINE-HF, NCT02653482), metabolic profiling of 234 participants revealed higher BHB levels in the DAPA group [132].

Post-analysis of the multicenter phase 3b randomized controlled trial EMMY (*Impact of EMpagliflozin on Cardiac Function and Biomarkers of Heart Failure in Patients with Acute MYocardial Infarction*, NCT03087773) revealed strong indirect evidence for the protective role of BHB upon iSGLT2 treatment. The EMMY study evaluated the effect of EMPA on acute MI, and a significant amelioration of cardiac function parameters was observed [133]. A post-analysis demonstrated that EMPA treatment was associated with higher levels of BHB [26]. Moreover, an increase in BHB levels during the study period was associated with better heart function and lower MI markers [26].

### 5.3. SGLT2 Inhibitors and Diabetes: Animal Studies

iSGLT2s protect against DKD by increasing endogenous ketone body levels, which counteract mTORC1 hyperactivation in damaged proximal tubules [134].

DAPA mitigated oxidative stress and cellular senescence by elevating BHB levels [135,136]. Specifically, in chronic heart failure murine models, DAPA protected against heart failure through macrophage inhibition rather than SGLT2 inhibition [137]. DAPA also had a positive effect on cardiac microvascular dysfunction and endothelial injury during ischemia–reperfusion injury (IRI) by inhibiting the XO-SERCA2-CaMKII-cofilin pathway. This led to improved endothelial barrier function, reduced apoptosis, and preserved microvascular integrity [138]. Furthermore, DAPA alleviated DKD and modulated gut microbiota over time, with improved gut flora composition and bile acid [139]. DAPA also supports human islet function in a hyperglycemic environment as it increases alpha and beta cell proliferation and reduces apoptosis in xeno-transplanted diabetic mice. It also potentially promotes alpha-to-beta cell transdifferentiation [140].

EMPA elevates circulating and myocardial ketone levels, thus improving cardiac function in non-diabetic mice with acute myocardial dysfunction [141]. EMPA is also able to reduce weight gain and insulin resistance by enhancing energy expenditure, shifting metabolism towards fat utilization, and promoting the browning of adipose tissue in obese mice. Additionally, in a recent study, EMPA mitigated inflammation by polarizing macrophages towards the anti-inflammatory M2 phenotype [142]. This drug protected the heart by suppressing autophagic cell death in cardiomyocytes by inhibiting Na+/H+ exchanger 1 (NHE1), reducing infarct size and myocardial fibrosis, and improving cardiac function in myocardial infarction models [143]. Furthermore, EMPA improved cardiac function and reduced ferroptosis, fibrosis, apoptosis, and inflammation in doxorubicin-treated mice via NLRP3- and MyD88-related pathways [144]. In diabetic mice, EMPA accelerated atherosclerosis regression by reducing plaque size and enhanced vascular health [116]. Moreover, it prevented diet-induced obesity, insulin resistance, and hepatic steatosis in mice, improving metabolic health and muscle mitochondrial morphology regardless of diet type [145]. EMPA reduced hyperuricemia by upregulating the uric acid transporter ABCG2 via AMPK/AKT/CREB signaling pathway, contributing to its therapeutic effects in T2D [146]. It also enhanced cardiac energy by increasing cytosolic and mitochondrial ATP levels, improving cardiac robustness, and aiding recovery from ischemia–reperfusion injury, which may contribute to its heart failure benefits in clinical trials [146]. Li et al. demonstrated that EMPA improved the structure and function of the diabetic heart, reduced oxidative stress, and mitigated myocardial fibrosis in T2D KK-Ay mice by inhibiting the TGF-β/Smad pathway and activating the NRF2/ARE signaling pathway [117]. NRF2 exacerbates DKD by upregulating certain genes, leading to lipid accumulation in renal proximal tubular cells. SGLT2 inhibition with EMPA improved endothelial function and reduced arterial stiffness in aged mice [147]. Another study conducted by Tan et al. demonstrated that EMPA elevates circulating and myocardial ketone levels, thus improving cardiac function in non-diabetic mice with acute myocardial dysfunction [141]. A combination of EMPA and metformin improved islet endothelial function and insulin secretion in diabetic mice, with EMPA showing superior effects and a combination of both drugs yielding the greatest benefit [148].

Ertugliflozin (ERTU) instead enhances left ventricular function and reduces fibrosis in a murine pressure overload model by increasing AMPK signaling and ketone body utilization while decreasing insulin signaling and mTOR pathway activation [149].

### 5.4. SGLT2 Inhibitors and Diabetes: Human Studies

iSGLT2s increase the plasma glucagon/insulin ratio, thus enhancing ketone body production in vivo, but this effect does not result from a direct action on human pancreatic islet cells [150]. As mentioned earlier, numerous clinical studies have reported that iSGLT2s elevate BHB levels. For instance, in a clinical trial conducted by Scarr et al., they reported that iSGLT2 increased BHB levels in serum and urine by upregulating ketogenic enzymes in various organs, with a more pronounced effect observed in women [151]. iSGLT2s elevated BHB levels and increased the risk of diabetic ketoacidosis, particularly in Type 1 Diabetes (T1D), although long-term use in T2D patients showed moderate increases in ketone levels related to cardiometabolic benefits [152,153].

iSGLT2s reduced diabetes-induced metabolic perturbations in kidney biopsies from treated patients by suppressing mTORC1 signaling, particularly affecting glycolysis and TCA cycle pathways [154]. Moreover, they reduce hyperglycemia and offer cardiovascular and kidney benefits potentially mediated by increased ketone levels, which have anti-oxidative and anti-inflammatory effects. However, these benefits come with a risk of ketoacidosis, particularly in T1D, highlighting a possible ‘double-edged sword’ effect of the drug class. Hence, they are best used in combination with other antihyperglycemic agents [155,156]. iSGLT2s have shown promising cardiac and renal protective effects, even in non-diabetic patients. They play a role in redox homeostasis and offer potential therapeutic benefits across various diseases, including liver diseases, neural disorders, and cancers [157]. Additionally, iSGLT2s showed potential as anti-aging therapies due to their anti-inflammatory and antioxidative effects, impacting pathways like AMPK/SIRT1/PGC-1α and reducing oxidative stress. Further human studies are needed to explore their role in age-related diseases and inflammation [158].

DAPA not only lowered blood glucose but also enhanced beta cell regeneration in T2D by promoting beta cell self-replication, alpha-to-beta cell conversion, and duct-derived beta cell neogenesis, partially mediated by GLP-1 [159].

EMPA reduced high glucose-induced inflammation and fibrosis in human kidney proximal tubular cells by inhibiting glucose transport and did not affect SGLT1/GLUT2 expression [160]. EMPA also significantly decreased liver fat without affecting myocardial fat [161]. Another study has shown that EMPA improved DKD by reducing mitochondrial fission via the AMPK/SP1/PGAM5 pathway, with effects diminished by AMPK inhibition [162,163]. Kim et al. demonstrated that EMPA exerts cardioprotective effects by mitigating NLRP3 inflammasome activation, increasing cardiac ketone oxidation, and decreasing serum insulin levels in T2D and CVD patients [73].

CANA reduced myocardial NADPH oxidase activity and improved nitric oxide synthase coupling via the SGLT1/AMPK/Rac1 signaling pathway, resulting in significant anti-inflammatory and anti-apoptotic outcomes in the human myocardium [164]. Moreover, CANA enhanced T cell-mediated cytotoxicity by promoting PD-L1 degradation, thus offering a potential strategy to overcome tumor immune evasion [165].

Matsubayashi and colleagues reported that tofogliflozin, another iSGLT2, significantly increases hepatic insulin clearance, reduces triglyceride levels, and elevates BHB levels, as well as contributes to hyperinsulinemia correction in T2D patients [166].

Interestingly, elevated serum BHB levels could potentially worsen the inflammatory immune response in patients with alopecia areata, with higher levels correlating with the severity of hair loss [167].

## 6. Conclusions and Future Directions

Ketone bodies represent natural compounds that our body produces to cope with stress conditions. In addition to their role as glucose-sparing energy carriers, ketones are involved in a variety of molecular signaling functions that may influence a broad range of human diseases [168,169]. Ketones are indeed essential for postnatal heart development; in fact, their deficiency impairs mitochondrial maturation and heart function [170].

In recent years, there has been considerable interest in these molecules as food supplements due to their potential for improved athletic performance and therapeutic glucose-lowering effects in people with diabetes. Of note, exogenous ketone supplements may be of particular interest for individuals living with T1D, by serving as an alternative fuel substrate to reduce the reliance on glucose utilization, sparing endogenous glycogen, and reducing the risk of hypoglycemia in certain situations, such as exercise. Moreover, recent studies raise the intriguing question of whether circulating ketone bodies may directly modulate immune cell function, providing a premise for future investigations into the potential role of these metabolites in other autoimmune diseases.

**Figure 1:**
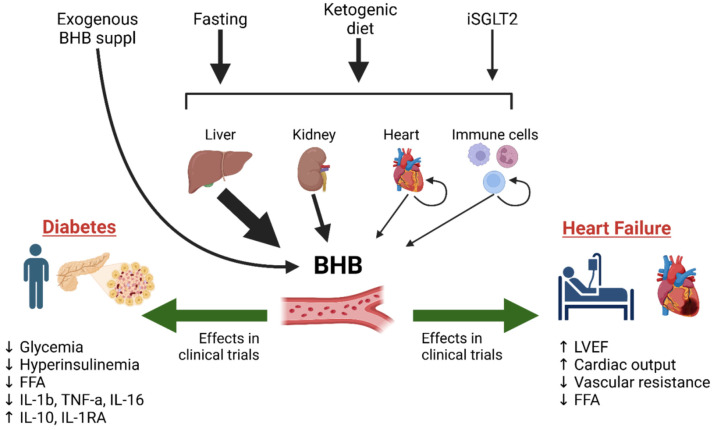
Ketone bodies are produced in the organism during carbohydrate or general nutrient insufficiency like fasting or ketogenic diet. Among all ketone bodies, β-hydroxybutyrate (BHB) is the most abundant and its levels in circulation are changing much faster. The predominant source of BHB is the liver. Under specific disease conditions, other organs, like kidneys and the heart, could produce substantial amounts of BHB. Inhibitors of Sodium-Glucose Transporter 2 (iSGLT2) were reliably demonstrated to promote ketogenesis. In addition to conventional sites of BHB production, iSGLT2 was found to promote ketosis in macrophages and CD8+ T cells. However, the role of unconventional sites of ketosis in whole-body BHB balance remains inconclusive. Exogenous supplementation of BHB is a highly efficient and safe way to increase circulating levels of BHB. In a series of clinical trials exogenous BHB supplementation was demonstrated to exert beneficial action on heart failure and diabetes.

Specifically, a functional role as a key signaling molecule is increasingly acknowledged for BHB, the most abundant ketone circulating in the human body. Low millimolar concentrations of BHB may improve endothelial function and vascular health by modulating inflammation, senescence, and metabolism [171]. Recently, we and others have reported that BHB attenuates maladaptive chromatin remodeling and mitochondrial dysfunction in postischemic heart failure, highlighting its potential as a therapeutic agent in cardiovascular health [42]. BHB modulates a number of signaling pathways with implications for several metabolic disease; interestingly, it is an endogenous inhibitor of many NAD+-independent HDACs, potentially having a direct effect on glucose homeostasis, oxidative stress, and mitochondrial function [90,172,173,174,175,176]. A graphical summary of ketone’s protective effects on heart and pancreatic beta cells is depicted in Figure 1.

Considering the tremendous translational potential of ketone bodies in CVD and diabetes, and the bottleneck in efforts to develop potential therapeutics in both fields, especially in elderly people, this area is worthy of attention and might significantly improve the lives of patients living with these diseases.
